# Intrapancreatic Gastric Duplication Cysts: A Report of Two Cases and a Systematic Review

**DOI:** 10.7759/cureus.87403

**Published:** 2025-07-07

**Authors:** Aleksandra I Sadecka, Iwona Rzewnicka

**Affiliations:** 1 Department of Pediatric Surgery, Medical University of Warsaw, Warsaw, POL

**Keywords:** abdominal cystic mass, foregut duplication cyst, intrapancreatic gastric duplication cyst, pancreatic cyst, pancreatitis

## Abstract

Duplications of the gastrointestinal tract are congenital anomalies characterized by a heterogeneous clinical presentation and challenging diagnostic and therapeutic processes. A systematic review was conducted on intrapancreatic gastric duplications in the pediatric population, and we present two clinical cases of patients with this condition. Intrapancreatic gastric duplication is an extremely rare congenital anomaly with variable clinical manifestations and complex management. However, it should be considered in the differential diagnosis of pancreatitis and abdominal masses in children.

## Introduction

Duplication of the alimentary tract is a rare congenital anomaly, with an estimated incidence of one in 4,500 live births [1]. The most common form involves the small intestine, while gastric duplications account for approximately 2-8% of cases [2]. The clinical presentation is variable and depends on the location and size of the cyst, the patient’s age, and the presence of associated comorbidities [2]. Among gastrointestinal duplications, intrapancreatic gastric duplications are particularly challenging to manage and may lead to life-threatening complications [3]. We present two cases of pediatric patients with this condition in this report. A systematic review was also conducted on intrapancreatic gastric duplications in the pediatric population. 

## Case presentation

Case 1

A one-year-old female patient was admitted to the Department of Pediatric Surgery for elective surgery due to a suspected gastric duplication. The patient presented with malnutrition and inadequate weight gain over the preceding six months. At the time of evaluation, she was being fed exclusively via a nasogastric tube. Her medical history was notable for an aberrant right subclavian artery (ARSA), two previous episodes of urinary tract infection, and iron deficiency anemia. Physical examination confirmed malnutrition, with no other significant abnormalities. 

**Figure 1:**
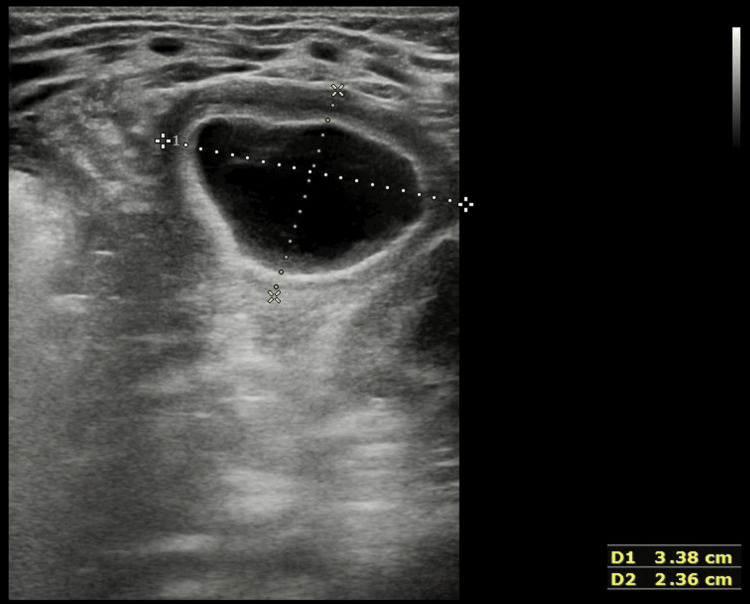
Abdominal ultrasound demonstrating a cystic lesion with a layered (stratified) wall located in the left epigastric region.

**Figure 2:**
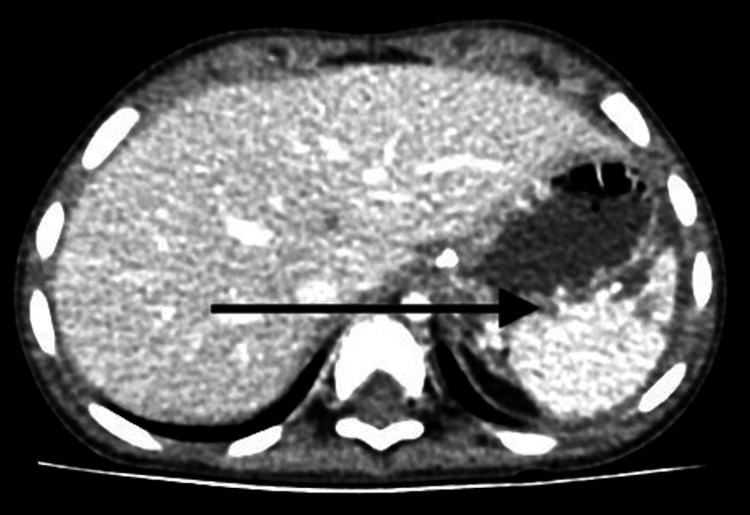
Contrast-enhanced CT demonstrating irregular, dilated, and tortuous vessels extending to the pancreatic tail (arrow).

Abdominal ultrasound revealed a 33 x 23 mm cyst with a stratified wall located in the left epigastric region, as well as another cyst, 65 mm in diameter, adjacent to the pancreatic tail (Figure 1). The head and body of the pancreas appeared heterogeneous and thickened, with indistinct borders. A contrast-enhanced computed tomography (CT) revealed multiple fluid collections within the intestinal mesentery, along with irregular, dilated, and tortuous vessels extending toward the pancreatic tail (Figure 2), raising suspicion of a vascular malformation. Further radiological assessment with abdominal magnetic resonance imaging (MRI) confirmed the presence of irregular peripancreatic fluid collections, an edematous pancreatic tail, and a thickened, tortuous pancreatic duct. Laboratory tests showed elevated fecal pancreatic elastase levels, while serum amylase and lipase levels remained within normal limits.

The patient underwent elective laparotomy. Intraoperatively, a cystic mass and inflammatory changes in the distal pancreas were identified, along with thrombosis of the splenic vein. A distal pancreatectomy and splenectomy were performed, and the cystic lesion was completely excised. Histopathological examination confirmed the diagnosis of a gastric duplication cyst located within the pancreas. The postoperative course was uneventful. 

Case 2 

**Figure 3:**
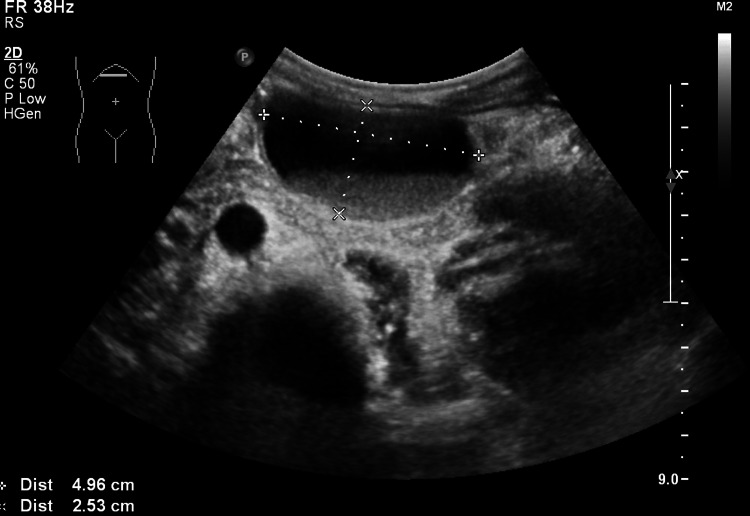
Abdominal ultrasound showing a thin-walled cyst with a sediment layer, measuring approximately 5 × 3 cm, located adjacent to the upper part of the pancreas at the junction of the body and tail.

**Figure 4:**
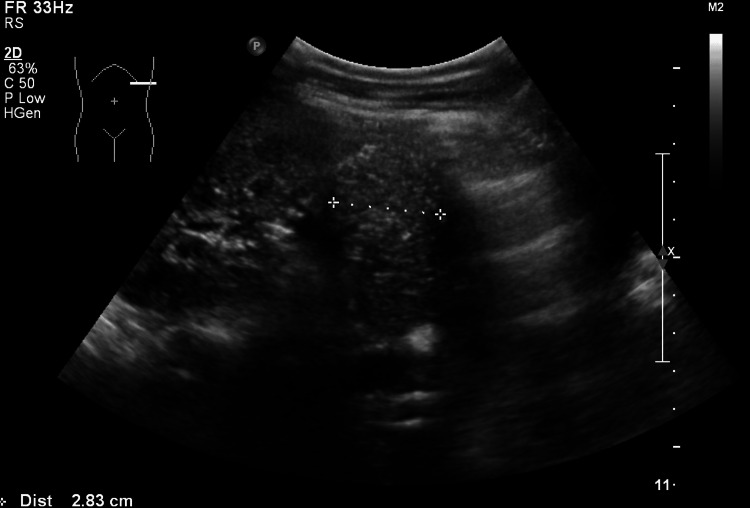
Abdominal ultrasound showing a cyst located within the pancreatic parenchyma.

**Figure 5:**
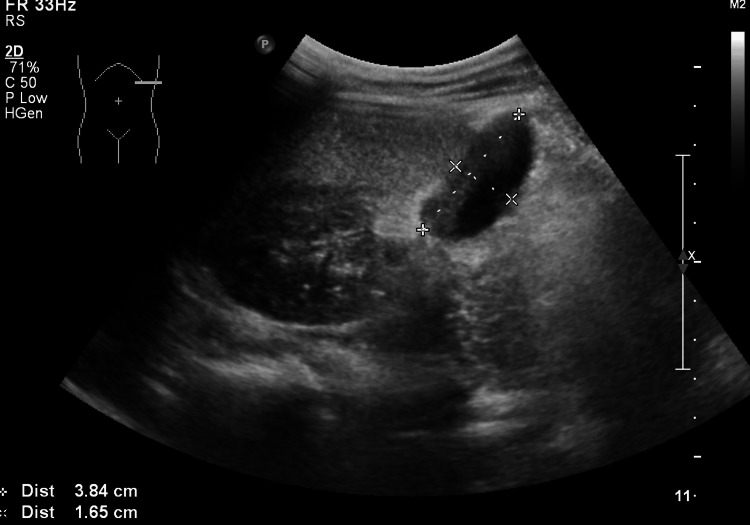
Abdominal ultrasound demonstrating a cyst located in the pancreatic tail.

An 11-year-old male patient was admitted to the Department of Pediatric Surgery due to a suspected pancreatic cyst. The lesion was incidentally detected on abdominal ultrasound and subsequently confirmed by contrast-enhanced abdominal CT. Ultrasound revealed thickening of the pancreatic duct and a thin-walled cyst with a sediment layer, measuring approximately 5x3 cm, located adjacent to the upper part of the pancreas at the junction of the body and tail (Figure 3). At the same level, a small cyst measuring 30×7 mm was identified within the pancreatic parenchyma (Figure 4). A third similar lesion, measuring 38×17 mm, was visualized in the region of the pancreatic tail (Figure 5).

The patient’s medical history included two prior incidents of lumbar trauma and a diagnosis of epilepsy. The patient was otherwise asymptomatic, and physical examination revealed no significant abnormalities.

The patient underwent surgery. Exploratory laparotomy revealed a gastric duplication cyst adjacent to the greater curvature of the stomach, measuring 10 cm in length, extensive intra-abdominal adhesions in the upper left epigastric region, and two pancreatic cysts located in the body and tail of the pancreas. The gastric duplication cyst was resected, and a distal pancreatectomy was performed. Histopathological examination confirmed the presence of three enteric-type cysts. The postoperative course was complicated by the development of a pancreatic fistula, which required reoperation and an additional distal pancreatic resection. 

## Discussion

Systematic review

Methods

**Figure 6:**
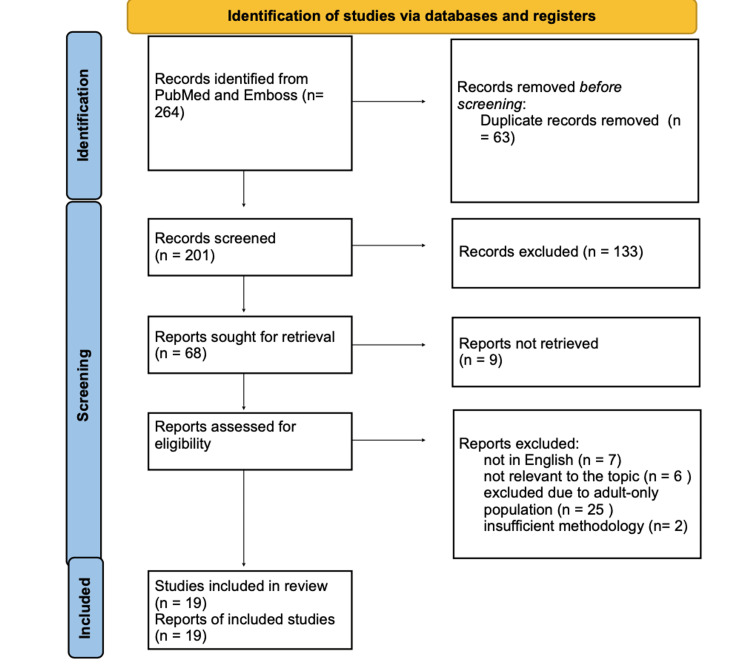
PRISMA 2020 flow diagram PRISMA: Preferred Reporting Items for Systematic reviews and Meta-Analyses

We conducted a systematic literature review in accordance with the Preferred Reporting Items for Systematic reviews and Meta-Analyses (PRISMA) 2020 guidelines. A comprehensive search was performed in the PubMed and Embase databases from 2000 to 2025 using the following keyword combination: (gastric duplication) AND (pancreas). The search yielded 264 records. Duplicate entries (n=63) were removed using Zotero (Corporation for Digital Scholarship, Corporation for Digital Scholarship, United States). The titles and abstracts of the remaining articles were independently screened by two reviewers for relevance to the research question, resulting in the exclusion of 133 records. Reports that could not be retrieved were excluded (n=9). The remaining full-text articles were assessed for eligibility. Studies were excluded if they were not in English (n=7), not relevant to the topic (n=6), focused exclusively on the adult population (n=25), or demonstrated insufficient methodology (e.g., abstracts only or letters to the editor) (n=2). Finally, a total of 19 studies were included in the qualitative synthesis. The study selection process is illustrated in the PRISMA 2020 flow diagram (Figure 6).

Results

The literature review on intrapancreatic gastric duplication included a total of 22 patients and indicates that this anomaly primarily affects female patients (15 females, seven males) and predominantly occurs in young children under the age of five (17 out of 22 patients). The most common presenting symptom was abdominal pain (10/22), followed by nausea, anorexia, feeding difficulties or appetite loss (7/23), weight loss or failure do gain weight (5/22), vomiting (3/22), and gastrointestinal (GI) bleeding (upper GI: 2/22, lower GI: 1/22). In the analyzed group, 11 patients presented with acute, acute-on-chronic, or recurrent pancreatitis. Physical examination was typically unremarkable; however, a palpable abdominal mass was noted in three patients, and two patients presented with abdominal guarding. Radiological evaluation included ultrasound in nearly all cases, supplemented by abdominal CT (12/22), MRI, or both. In some cases, detailed imaging of the biliary system was performed using magnetic resonance cholangiopancreatography (MRCP) or endoscopic retrograde cholangiopancreatography (ERCP) (8/22). Three patients were diagnosed antenatally via prenatal ultrasound. Ultrasound was particularly useful in some cases where follow-up imaging revealed changes in the shape and size of the cyst, features characteristic of gastrointestinal duplications. Surgical treatment included cystectomy (11/22), distal pancreatectomy (5/22), resection of the gastric duplication cyst with duplicated pancreatic tissue (2/22), and cystojejunostomy with Roux-en-Y anastomosis (1/22). One patient did not undergo surgery, and in three cases, surgical details were not reported. Laparotomy was the predominant surgical approach, with laparoscopy performed in only three cases. Gastric duplication cysts were associated with pancreatic abnormalities (either duplication of pancreatic tissue or duct) in eight cases. The results are summarized in the table (Table 1).

**Table 1:** Intrapancreatic gastric duplication: clinical features and management US: abdominal ultrasound examination; MRCP: magnetic resonance cholangiopancreatography; ECPW: endoscopic retrograde cholangiopancreatography; CT: computed tomography; EUS: endoscopic ultrasound; MRI: magnetic resonance imaging; M: male; F: female

Authors, year	Sex and age of patient	Symptoms	Imaging	Localization in the pancreas	Duplication of the pancreatic duct	Connection to the pancreatic duct/gastric lumen	Pancreatitis	Surgery
1. Agrawal et al. (2024) [4]	F, 2 years	hematemesis	US, MRCP, CT, upper GI series	tail	(-)	(-)	(+)	resection of the cyst and incomplete annular pancreas
2. Andronikou et al. (2002) [5]	F, 3 years	recurrent abdominal pain, nausea	US, CT	tail	(-)	(-)	(-)	dystal pancreatic resection
3. Brown (2023) [6]	M, 1 year	-	US, MRI	head	(-)	(-)	(-)	cystectomy
4. Brown et al. (2023) [6]	F, 6 months	-	US, CT, MRI	tail	(-)	(-)	(-)	cystectomy
5. Cadili et al. (2022) [7]	F, 8 months	upper GI bleed	Endoscopy, US, MRI	head	(-)	(-)	(+)	cystectomy
6. Gorsler et al. (2008) [8]	F, 1 year	6-week weight gain failure	US	body	(-)	(-)	(-)	cystectomy
7. Gugig et al. (2004) [9]	F, 7 years	abdominal pain, weight loss, bloating	US, CT, MRI, ERCP	tail	(+)	(-)	(+)	distal pancreatectomy
8. Hishiki et al. (2008) [10]	M, 1 month	projectile vomiting, weight loss	US	head	(-)	connection to the pancreatic duct	(-)	1.pyloromyotomy, biopsy, 2. cystectomy
9. Jain et al. (2015) [11]	F, 5 years	recurrent upper abdominal pain	US, MRI, CT, MRCP	connected to pancreatic duplication	(+)	connection to the gastric lumen	(+)	resection of the GD with adjacent pancreatic duplication
10. Kohno et al. (2010) [12]	F, 3 years	abdominal pain	US, CT, MRCP	tail	(+)	(-)	(-)	cystectomy
11. Komori et al. (2013) [13]	F, 1 year	abdominal pain, lack of petite, fever	US, CT, MRCP	body with pancreatic duplication	(-)	(-)	(+)	cystojejunostomy Roux en Y
12. Kumar et al. (2023) [14]	F, 7 years	recurrent paraumbilical pain	US, CT, MRCP	neck with pancreatic duplication	(+)	(-)	(+)	no operation
13. Lee et al. (2014) [15]	M, 2 years	abdominal pain, anorexia, weigth loss	CT	tail	(-)	(-)	(+)	1. biopsy 2.diatal pancreatectomy
14. Lee et al. (2011) [16]	F, 2 years	poor feeding, abdominal pain	US, CT	body	(-)	communication with the pancreatic duct	(+)	cystectomy
15. Lee et al. (2011) [16]	F, 10 months	no symptoms	US, MRI	tail	(-)	communication with the pancreatic duct	(-)	cystectomy
16. Lee et al. (2011) [16]	F, 8 months	poor feeding and failure to thrive	US, upper GI endoscopy	body	(-)	(-)	(-)	cystectomy
17. Miyamoto et al. (2024) [17]	M, 12 years	recurrent acute pancreatitis	CT, MRI, MRCP, ECPW, EUS	tail	(+)	(-)	(+)	distal pancreatectomy
18. Nakazawa et al. (2005) [18]	M, newborn	no symptoms	US, CT, X-ray	body	(-)	(-)	(-)	cystectomy
19. Pina-Prata et al. (2023) [19]	F, 5 months	vomiting		tail duplication		(-)	(-)	
20. Rao et al. (2023) [3]	M, 8 months	poor feeding , rectal bleeding	US (neg.), upper and lower GI endoscopy (neg.)	body and tail	(-)	(-)	(-)	exploratory laparotomy, distal pancreatectomy, splenectomy, ileal resection
21. Shabtaie et al. (2017) [20]	M, 6 years	recurrent abdominal pain	US, CT, MRI	head with pancreatic duplication	(-)	(-)	(+)	cystectomy
22. Webster et al. (2001) [21]	F, 17 years	abdominal pain, anorexia	us, CT, MRCP, ECPW	tail	(+)	communication with the pancreatic duct	(+)

Discussion

The demographics of patients diagnosed with intrapancreatic gastric duplication cysts vary among authors. In our review, we identified a higher number of female patients, most of whom were under five years of age; however, some studies report a male predominance in this condition [22]. Radiological assessment is typically initiated with ultrasound in patients presenting with abdominal symptoms, although an increasing number of asymptomatic cases are diagnosed incidentally, or even via prenatal ultrasound [6,18]. 

Abdominal pain of varying intensity and nutritional symptoms, including vomiting, nausea, and appetite loss, are typical clinical features of intrapancreatic gastric duplication. These manifestations may indicate underlying pancreatitis, which was commonly observed in the reviewed cases [4,9,11,13-17,20,21]. However, an intrapancreatic gastric duplication cyst may also be asymptomatic and discovered incidentally, as was the case in our patient. 

Intrapancreatic gastric duplication cysts can be detected using various radiological modalities. With the advancement of prenatal care, some cases are now diagnosed during routine prenatal ultrasound examination [6,18]. In our patients, as well as in the literature review, the initial detection of the cyst was typically made via abdominal ultrasound. Subsequent diagnostic evaluation focused on characterizing the lesion and assessing its relationship to the biliary duct system and the gastrointestinal tract lumen, which was based primarily on CT scans, in some cases complemented by MRI. In only about one-third of cases was it necessary to perform MRCP or ERCP. Although specific pediatric guidelines for the diagnostic evaluation of pancreatic cysts are lacking, Elta et al. have published general recommendations for the adult population [23]. They recommend MRI or MRCP as a primary imaging modality, with CT and EUS (endoscopic ultrasound) as alternatives in patients unable to undergo MRI.

Surgical treatment of intrapancreatic duplication cyst typically involves cystectomy, which was feasible in most reported cases (Table 1). In some patients, distal pancreatectomy was necessary, while more invasive procedures such as cystojejunostomy were rarely required. In our patients, distal pancreatectomy was performed. The extent of the procedure was determined by the location of the duplication cyst within the pancreas and the inability to safely enucleate the lesion without risking damage to the pancreatic parenchyma. No postoperative complications were reported in the reviewed cases, apart from one patient treated at our institution and presented in our report, who required reoperation due to a pancreatic fistula. However, follow-up data were available for only eight patients, with durations ranging from six months to four years (average: 15 months) [3,6,9-11,15,17,18]. A laparoscopic approach was rarely employed but was reported as feasible in three cases [12,16,19]. 

Pancreatitis was a predominant clinical manifestation in half of the analyzed cases, and gastric duplication cysts were associated with pancreatic abnormalities or obstruction of the pancreatic duct in many of them (Table 1). Suzuki et al. described the main characteristics of pancreatitis in children, identifying obstruction of the pancreatic duct, caused, among other factors, by tumors, as a key risk factor [24]. However, intrapancreatic gastric duplication cysts were not mentioned in their report. Similarly, Pohl et al. reported that obstructive factors are responsible for 10-30% of pediatric pancreatitis cases and contribute to both recurrent and chronic forms of the disease [25]. Our reported cases supports these findings and underscores the importance of radiological evaluation in the assessment of pancreatitis in children.

## Conclusions

Intrapancreatic gastric duplication is an extremely rare congenital anomaly with variable clinical manifestations and complex management. However, it should be considered in the differential diagnosis of pancreatitis and abdominal masses in children. Moreover, surgical intervention remains the treatment of choice and should be preceded by thorough radiological evaluation.

We believe that the diagnostic and therapeutic approach used in our department, consisting of initial ultrasound evaluation, detailed preoperative assessment with contrast-enhanced CT, and surgical treatment adapted to the location of the lesion, most commonly involving distal pancreatectomy, is appropriate.

## References

[1] Schalamon J, Schleef J, Höllwarth ME (2000). Experience with gastro-intestinal duplications in childhood. Langenbecks Arch Surg.

[2] Zhang L, Chen Q, Gao Z, Xiong Q, Shu Q (2017). Diagnosis and treatment of gastric duplication in children: a case report. Exp Ther Med.

[3] Rao KL, Sunil I, Pimpalwar A, Vaiphei K, Chowdhary S (2003). Intrapancreatic gastric duplication cyst presenting as lower gastrointestinal bleeding. J Pediatr Surg.

[4] Agrawal P, Bhattar K, Rojas C, Diaz D (2024). Enteric duplication cysts: an overlooked cause of recurrent pancreatitis. Am. J. Gastroenterol.

[5] Andronikou S, Sinclair-Smith C, Millar AJ (2002). An enteric duplication cyst of the pancreas causing abdominal pain and pancreatitis in a child. Pediatr Surg Int.

[6] Brown A, Rust D, Chwals W (2023). Heterotopic duplication cysts. J Pediatr Surg Case Rep.

[7] Cadili L, Cullen KL, Finn NJ, Singh A, Webber E, Hayashi AH (2022). A rare case of a congenital pancreatic duplication cyst in an infant complicated by an upper GI bleed, pancreatitis, cyst infection and gastric outlet obstruction. J Surg Case Rep.

[8] Gorsler CM, Rodriguez R, Kistler W, Baumgartner G (2008). Coincidence of a ductal pancreatic cyst and a gastric duplication cyst: a case report. Eur J Pediatr Surg.

[9] Gugig R, Ostroff J, Chen YY, Harrison M, Heyman MB (2004). Gastric cystic duplication: a rare cause of recurrent pancreatitis in children. Gastrointest Endosc.

[10] Hishiki T, Saito T, Terui K, Mitsunaga T, Nakata M, Matsuura G, Yoshida H (2008). A rare presentation in a case of gastric duplication cyst communicating to the pancreatic duct: coincidental detection during pyloromyotomy for hypertrophic pyloric stenosis. J Pediatr Surg.

[11] Jain AS, Patel AM, Jain SR, Thakkar A (2015). Accessory pancreatic lobe with gastric duplication cyst: diagnostic challenges of a rare congenital anomaly. BMJ Case Rep.

[12] Kohno M, Ikawa H, Konuma K (2010). Laparoscopic enucleation of a gastroenteric duplication cyst arising in a pancreatic tail that did not communicate with the pancreatic duct: report of a case. Surg Today.

[13] Komori K, Hirobe S, Toma M, Nishimura G, Fukuzawa R (2013). A gastric duplication cyst of the pancreas associated with a bifid tail causing pancreatitis. J Pediatr Surg Case Rep.

[14] Kumar I, Meena S, Singh PK, Aggarwal P, Verma A (2024). A rare cause of acute recurrent pancreatitis in a child. Indian J Pediatr.

[15] Lee CL, binti Che Daud CZ, binti Ismail R (2014). Intrapancreatic gastric duplication cyst-a rare cause of chronic abdominal pain in childhood. J Clin Ultrasound.

[16] Lee TC, Kim ES, Ferrell LB (2011). Gastric duplication cysts of the pancreas: clinical presentation and surgical management. Eur J Pediatr Surg.

[17] Miyamoto S, Ishii Y, Serikawa M (2024). A case of young male with recurrent acute pancreatitis caused by an intrapancreatic gastric duplication cyst. Clin J Gastroenterol.

[18] Nakazawa N, Okazaki T, Miyano T (2005). Prenatal detection of isolated gastric duplication cyst. Pediatr Surg Int.

[19] Pina-Prata R, Rosa F, Fontes I, Morão S (2023). Gastric duplication cyst of a bifid pancreas: cause of recurrent vomiting. BMJ Case Rep.

[20] Shabtaie SA, Infante JC, Danton G, Neville HL, Perez EA, Sola JE, Hogan AR (2017). Accessory pancreatic lobe in association with a gastric duplication cyst. J Pediatr Surg.

[21] Webster J, Terry S, Humphrey D, Khan SA (2001). Anorexia and pancreatitis associated with a gastric duplication cyst of the pancreas. Surgery.

[22] Puligandla PS, Nguyen LT, St-Vil D, Flageole H, Bensoussan AL, Nguyen VH, Laberge JM (2003). Gastrointestinal duplications. J Pediatr Surg.

[23] Elta GH, Enestvedt BK, Sauer BG, Lennon AM (2018). ACG clinical guideline: diagnosis and management of pancreatic cysts. Am J Gastroenterol.

[24] Suzuki M, Minowa K, Isayama H, Shimizu T (2021). Acute recurrent and chronic pancreatitis in children. Pediatr Int.

[25] Pohl JF, Uc A (2015). Paediatric pancreatitis. Curr Opin Gastroenterol.

